# The COVID - Curated and Open aNalysis aNd rEsearCh plaTform (CO-CONNECT).

**DOI:** 10.23889/ijpds.v7i3.1792

**Published:** 2022-08-25

**Authors:** Emily Jefferson, Aziz Sheik, Susan Hopkins, Philip Quinlan

**Affiliations:** 1 University of Dundee; 2 University of Edinburgh; 3 UK Health Security Agency (UKHSA); 4 University of Nottingham

## Objectives

CO-CONNECT is making UK COVID-19 data Findable, Accessible, Interoperable and Reusable (FAIR) through a federated platform, which supports secure, anonymised research at scale and pace. This interdisciplinary project, spanning 22 organisations, is connecting data from >50 large research cohorts and data collected through routine healthcare provision across the UK.

## Approach

Across the UK, data has been collected that can help us answer key questions about COVID-19. As the data are in many places with many different processes it is difficult and complex for public health groups, researchers, policymakers, and government to find and access lots of high-quality data quickly and efficiently to make decisions. In collaboration with Health Data Research UK, CO-CONNECT is streamlining processes of accessing data for research.

## Results

1) Discovering data and meta-analysis: CO-CONNECT enables researchers to determine how many people meet their research criteria within the various datasets across the UK through the Health Data Research Innovation Gateway Cohort Discovery tool e.g. “How many people in each dataset have had a PCR test which was positive and were under the age of 40?” Only summary level, anonymous data are provided so researchers can answer such questions rapidly without requiring multiple data governance permissions and directly contacting each data source. The tool also supports aggregate level meta-analysis of the data.

2) Detailed analysis: With data governance approvals, researchers can analyse detailed level, standardised, linked, pseudonymised data in a Trusted Research Environment. The common format reduces the effort on each research project, supporting rapid research.

## Conclusion

Providing data in this de-identifiable, safe way enables rapid, robust research e.g., COVID-19 results from a test centre can be linked to hospital records along with prescriptions from pharmacies enabling researchers to understand whether people with different existing health conditions are more or less susceptible to COVID-19. If you want to know more visit https://co-connect.ac.uk

